# Large-scale evaluation of HIV-1 DNA drug resistance testing as a robust tool for clinical decision-making: A nationwide study in China

**DOI:** 10.1016/j.jpha.2025.101513

**Published:** 2025-12-09

**Authors:** Caihong Wu, Limin Zhang, Zhong Chen, Wencui Ma, Yanhua Fu, Ke Yang, Mei Liu, Yanjun Li, Xiaohong Chen, Mingjie Hou, Min Liu, Aihua Deng, Qingxia Zhao, Lukun Zhang, Quan Wang, Jun Peng, Yongli Li, Keji Deng, Jingsong Bai, Hai Long, Yaokai Chen, Hui Wang, Yun He, Jin Li, Jiahui Guo, Bianchuan Cao, Yizhi Cui, Min Wang, Tuofu Zhu, Jun Yao, Tong Wang

**Affiliations:** a State Key Laboratory of Bioactive Molecules and Druggability Assessment, Center of Clinical Laboratory, The First Affiliated Hospital, College of Life Science and Technology, Jinan University, Guangzhou, 510632, China; b Department of Infectious Diseases, Institute of HIV/AIDS, The First Hospital of Changsha, Changsha, 410005, China; c Department of Infectious Disease, Guiyang Public Health Treatment Center, The Affiliated Hospital of Guizhou Medical University, Guiyang, 550001, China; d Department of Antiviral Therapy, The First People's Hospital of Yuexi County, Liangshan, Sichuan, 615000, China; e Department of Infectious Diseases, Guangxi AIDS Clinical Treatment Center, The Fourth People's Hospital of Nanning, Nanning, 530023, China; f Department of Infectious Diseases, The Fourth Affiliated Hospital of Harbin Medical University, Harbin, 150001, China; g Department of Infectious Diseases, Henan Infectious Disease Hospital, Zhengzhou, 450015, China; h Department of Infectious Diseases, Chongqing Public Health Medical Center, Chongqing, 400036, China; i Jiangxi Chest Hospital, Nanchang, 330000, China; j The Third People's Hospital of Shenzhen, Shenzhen, 518112, China; k Department of Laboratory Medicine, Xinjiang Uygur Autonomous Region Infectious Disease Hospital, Urumqi, 830000, China; l Guangzhou SupBio Bio-Technologies and Science Co., Ltd., Guangzhou, 510530, China; m Dongguan Institute of Microscale and Precision Medical Measurement Co., Ltd., Dongguan, Guangdong, 523808, China; n Department of Infectious Diseases, The Third People's Hospital of Kunming, Kunming, 650500, China; o Department of Infectious Disease, The Ninth People's Hospital of Dongguan, Dongguan, Guangdong, 523076, China; p Department of Infectious Disease, The Affiliated Hospital of Southwest Medical University, Luzhou, Sichuan, 646000, China; q National Center for AIDS/STD Control and Prevention, Chinese Center for Disease Control and Prevention, Beijing, 102206, China

**Keywords:** Drug resistance mutations, Sanger sequencing, HIV-1 DNA, Reproducibility, Dominant sequence

## Abstract

Human immunodeficiency virus type 1 (HIV-1) drug resistance remains a major challenge in HIV/AIDS management, particularly in individuals with low-level viremia (LLV) where RNA-based drug resistance testing (DRT) often fails. Although HIV-1 DNA DRT represents a promising alternative, its clinical utility has been constrained by insufficient evidence. This nationwide study in China enrolled 9,428 people living with HIV (PLWH), analyzing 10,903 samples spanning a wide viral load (VL) spectrum. To improve RNA detection, an optimized primer design combined with an extracellular particle (EP)-HIV co-isolation technique was developed. We then evaluated the reproducibility of drug resistance mutation (DRM) profiles between paired RNA and DNA DRTs using Sanger sequencing (SS), with single-molecule sequencing employed to establish a dominant sequence threshold. Our findings demonstrated that primer optimization and EP-co-isolation significantly enhanced RNA amplification success. DRMs were prevalent across all VL strata. The combined concordance and degeneracy rates (C/D rates) (where multiple DNA DRMs included all RNA-derived DRMs) between RNA and DNA DRTs ranged from 90.4% to 100% in different gene regions, with higher discordance rates observed in the nucleoside reverse transcriptase inhibitor (NRTI) and non-NRTI (NNRTI) regions. Based on Stanford penalty scores across 25 antiretroviral drugs, the degeneracy group showed a 98.3% ± 1.7% interpretation agreement. Even within the discordance group, mean agreement remained high (89.5% ± 5.0%), with only four NNRTIs exhibiting agreement below 85%. The dominant sequence proportion threshold for HIV-1 DNA was determined to be 24.6%. This study provides strong evidence supporting the integration of HIV-1 DNA DRT into clinical practice for reliable drug resistance surveillance and treatment monitoring.

## Highlights

• 10,903 blood samples tested to justify clinical significance of HIV DNA DRT.
• EP-HIV co-isolation remarkably increases LLV DRT sensitivity.
• HIV RNA/DNA DRTs are highly reproducible across diverse VL states.
• Dominant threshold of 24.6 % for HIV-1 DNA SS is defined experimentally.
• DNA DRT can be employed as a cost-effective alternative to RNA DRT.

## Graphical abstract

**Figure:**
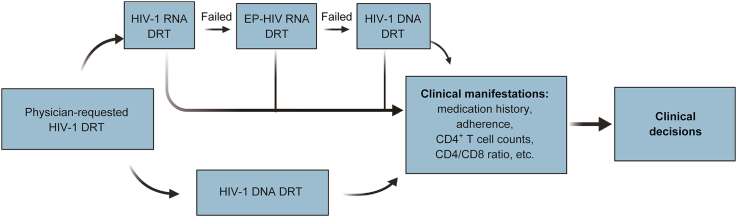

## 1 Introduction

Since the implementation of antiviral therapy (ART) for human immunodeficiency virus type 1 (HIV-1) over the past 40 years, HIV-1 drug resistance (HIVDR) has emerged as a profound public health threat in people living with HIV (PLWH). HIV-1 drug resistance mutations (DRMs) can be acquired through a complex array of mechanisms, giving rise to acquired HIVDR. These mechanisms include spontaneous mutations, viral recombination, host factor introduction, and selective pressure of antiretroviral treatment (ART) drugs [1]. Transmission of these drug-resistant HIV-1 strains to treatment-naïve PLWHs results in pandemics of pre-treatment HIVDR (PDR). The overall prevalence of PDR to any drug class varies across different countries, ranging from 2.8% to 11.4% [2].

In numerous guidelines, such as the National AIDS Testing Technology Standard of China (version 2020), a plasma/serum viral load (VL) of 1,000 copies/mL is designated as the threshold for PLWHs, who require standardized genotypic drug resistance testing (DRT) [3]. However, approximately 20% of chronically infected PLWHs experience persistent low-level viremia (LLV), characterized by a VL ranging from 50 to 1,000 copies/mL according to the World Health Organization (WHO) guideline [3,4]. A certain proportion of these PLWHs with LLV can be classified as treatment-failed based on numerous guidelines. For example, VL > 50 copies/mL and persistent VL > 200 copies/mL are defined as treatment failure by the European AIDS Clinical Society (EACS) [5] and the Chinese Medical Association [6], respectively. Notably, these individuals with LLV face elevated risks of developing non-AIDS-defining co-morbidities, including cancer, kidney diseases, and cardiovascular diseases [7]. Furthermore, studies have demonstrated that replication-competent HIV-1 can be recovered from plasma of PLWHs with VL < 50 copies/mL [8], and HIV DRMs are detectable in these samples [9]. As such, HIV DRTs are highly demanded by physicians for all treatment-naïve PLWHs, as well as for those who exhibit treatment failure, LLV or detectable residual viruses [10,11].

HIV DRT at RNA level (RNA DRT) using Sanger sequencing (SS) is a standard method for detecting HIV DRMs [1,3,12]. However, a significant proportion of physician-requested HIV DRTs fail to amplify plasma HIV-1 RNA, particularly in patients with LLV. For example, Yuan et al. [13] have reported that out of 345 LLV patients from China, 242 can be successfully amplified, resulting in an overall success rate of 70.1%. When RNA DRT fails, HIV-1 DNA based DRT (DNA DRT) has been proposed as an alternative. Nevertheless, its clinical significance remains uncertain, leading to caution in its application [5,14,15]. For instance, the U.S. Department of Health and Human Services Guideline (2024) states that DNA DRT may provide information about previously circulating resistant viral variants; however, the clinical application of HIV-1 proviral DNA assays has not been fully established, necessitating careful interpretation of DNA DRT results [15].

To address concerns regarding DNA DRT, the consistency of DNA DRT results with RNA DRT has been recognized as a prerequisite for its application [[16], [17], [18]]. Geretti et al. [17] found a high concordance rate of 97.8% (*n* = 91) between peripheral blood mononuclear cells (PBMCs) and plasma DRM profiles in a retrospective study of patients with a median VL of 4.8 log10 (namely 10^4.8^, or 6.3 × 10^4^) copies/mL. Similarly, Ma et al. [18] reported a concordance rate of 92.4% (*n* = 66) among treatment-naïve patients. However, Geretti et al. [14] have argued that most of these studies have small sample sizes and focus primarily on high VL samples, which limits their ability to comprehensively and objectively reflect the DRM concordance of DNA and RNA DRTs in diverse patient populations.

The uncertainty of promoting DNA DRTs in clinical practice largely stems from limitations in existing studies, including small sample sizes and a primary focus on individuals with high VLs. To address these knowledge gaps, this study employs a cross-sectional design with physician-requested samples including three distinct VL categories: VL > 1,000 copies/mL, LLV, and samples with detectable residual viruses (VL < 50 copies/mL). We improved the success rates of RNA DRT with optimized primers and/or an extracellular particle (EP)-based co-isolation method. This study systematically analyzed the reproducibility of DRM detection between RNA and DNA DRTs. Furthermore, with the single molecule sequencing (SMS) analysis, we determined the proportion cut-off of dominant sequences in SS to understand such reproducibility.

## 2 Methods

### 2.1 Ethics

This research was coordinated by The First Affiliated Hospital, Jinan University and The First Hospital of Changsha in collaboration with other institutions. This study was approved by the Ethics Review Board of the First Hospital of Changsha, China (Approval No.: 2021-028) and was performed following the Declaration of Helsinki. Digitally signed informed consents were obtained from all study participants prior to their involvement. To ensure participant privacy and data confidentiality, the following measures were implemented: i) all personally identifiable information was collected using unique study codes instead of direct identifiers and was stored separately from the research data; ii) electronic data were encrypted during storage and transmission, with access restricted to authorized researchers; and iii) the informed consent process explicitly detailed the data usage scope, confidentiality measures, and participants' right to withdraw without penalty. Participant information was kept strictly confidential in compliance with these protocols.

### 2.2 Participants and sampling

**Fig. 1:**
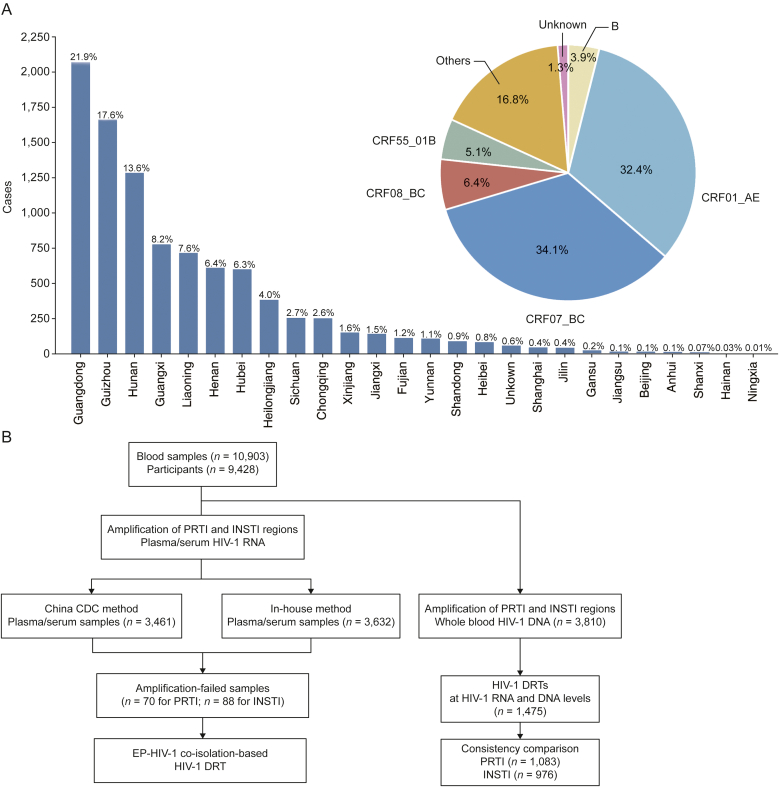
Participant characteristics and study design. (A) Geographical distribution and human immunodeficiency virus type 1 (HIV-1) subtype composition among participants. (B) Workflow for drug resistance testing (DRT) comparisons. PRTI: protease and reverse transcriptase inhibitor; INSTI: integrase strand transfer inhibitor; China CDC: Chinese Center for Disease Control and Prevention; EP: extracellular particle.

For physician-requested HIV-1 DRTs, blood samples were collected from 9,428 PLWHs across 25 provinces in China between January 2021 and September 2023 (Fig. 1A). In total, 10,903 peripheral blood samples were analyzed, including both plasma/serum and whole blood collected. Plasma/serum was prepared by centrifugation within 24 h post-blood draw, and all samples were stored at −80 °C until nucleic acid extraction to minimize pre-analytical variations. The most prevalent HIV-1 subtypes among the study participants were CRF01_AE (32.4% (3053/9428)) and CRF07_BC (34.1% (3210/9428)) (Fig. 1A), consistent with previous findings in Chinese populations [19].

### 2.3 Experimental settings

For the plasma/serum samples in this study, we compared two RNA DRT methods. A total of 3,461 samples were analyzed using the standardized Chinese Center for Disease Control and Prevention (China CDC) method [20,21], while 3,632 samples were subjected to an in-house method (Fig. 1B). Notably, for plasma/serum samples that were unsuccessful with both methods, we further utilized the EP-HIV-1 co-isolation-based approach to perform additional RNA DRT (Fig. 1B). DNA DRT was performed on 3,810 whole blood samples. Among these, 1,475 samples also had corresponding RNA DRT results, enabling a comprehensive comparison (Fig. 1B).

### 2.4 HIV-1 nucleic acid extraction and quantitation

As we previously described [22], HIV-1 RNA was extracted using Nucleic Acid Extraction or Purification Kits (SUPI-1010-1; SupBio Bio-Technologies and Science Co., Ltd., Guangzhou, China), and quantified with the HIV-1 Nucleic Acid Detection Kits (SupBio Bio-Technologies and Science Co., Ltd.). Similarly, HIV-1 DNA was extracted from 200 μL of whole blood samples with the Nucleic Acid Extraction or Purification Kits (SUPI-1015; SupBio Bio-Technologies and Science Co., Ltd.), and the HIV-1 DNA load was quantified with HIV-1 DNA Quantitative Detection Kits (SupBio Bio-Technologies and Science Co., Ltd.) (limit of quantification (LOQ) = 100 copies/10^6^ cells).

### 2.5 DRT for HIV-1

We compared the performance of China CDC with in-house methods regarding polymerase chain reaction (PCR) amplifications of the protease and reverse transcriptase inhibitor (PRTI) and integrase strand transfer inhibitor (INSTI) regions within the HIV-1 *pol* gene. For the China CDC method, the primers and amplification procedures for the PRTIs and INSTIs regions can be respectively found in Chen et al. [21] and Liu et al. [20]. The in-house method was performed as we previously described [22]. Sequences of the optimized PCR primers can be found in Table S1 (Patent Nos.: CN116426688A and CN116411134A). All sequences reported in this study were uploaded in the HIV Gene Sequences Database, China (https://nmdc.cn/hiv/) (Submission No.: SUB1720661380069). To identify DRMs, sequences were analyzed through the Stanford University HIV Drug Resistance Database (https://hivdb.stanford.edu/hivdb/by-patterns). Sequences with DRMs were determined to be susceptible (<15) or resistant (≥15) based on their Stanford penalty scores against 25 ART drugs. These drugs included protease inhibitors (PIs): atazanavir/ritonavir (ATV/r), darunavir/ritonavir (DRV/r), lopinavir/ritonavir (LPV/r), fosamprenavir/ritonavir (FPV/r), indinavir/ritonavir (IDV/r), nelfinavir (NFV), saquinavir/ritonavir (SQV/r), and tipranavir/ritonavir (TPV/r); nucleoside reverse transcriptase inhibitors (NRTIs): abacavir (ABC), zidovudine (AZT), emtricitabine (FTC), lamivudine (3TC), tenofovir disoproxil fumarate (TDF), stavudine (D4T), and didanosine (DDI); non-NRTIs (NNRTIs): doravirine (DOR), efavirenz (EFV), etravirine (ETR), nevirapine (NVP), and rilpivirine (RPV); and INSTIs: bictegravir (BIC), cabotegravir (CAB), dolutegravir (DTG), elvitegravir (EVG), and raltegravir (RAL).

### 2.6 EP-HIV-1 co-isolation

As we previously described [23], the serum/plasma samples were subjected to EP isolation with a medical device developed by our group (SupBio Bio-Technologies and Science Co., Ltd.) (Certificate No.: 20170482). Briefly, 750 μL of serum/plasma was mixed with 1,875 μL of buffer A and 37.5 μL of buffer B (provided with the device) and incubated for 10 min at room temperature. The mixture was then loaded into an ultrafiltration device and centrifuged at 1,000 *g* for ∼20 min until the retentate was concentrated to approximately 300 μL. This retentate, designated as the EP isolate, was aliquoted and stored at −80 °C until use.

The immunoblotting (IB) analysis was performed as we previously described with minor modifications [24]. Specifically, the EP isolates were treated with the lysis buffer (1% sodium dodecyl sulfate (SDS), 10 mM phenylmethylsulfonyl fluoride (PMSF), 1× PI), and 40 μg protein extract was loaded for each IB analysis. The primary antibodies included rabbit anti-HIV-gp120 monoclonal antibody (mAb) (1:10,000, Cat. No.: 11233-RP02; Sino Biological, Beijing, China), mouse anti-apoprotein B (APOB) mAb (1:20,000, Cat. No.: sc-393636; Santa Cruz Biotechnology, Dallas, Texas, USA), rabbit anti-integrin β3 mAb (1:1,000, Cat. No.: 13166; Cell Signaling Technology, Inc., Danvers**,** MA, USA), and rabbit anti-albumin mAb (1:2,000, Cat. No.: ab207327; Abcam, Cambridge, MA, USA). The secondary antibodies included horseradish peroxidase (HRP)-conjugated anti-rabbit (1:1,0000, Cat. No.: 7074; Cell Signaling Technology, Inc.) and anti-mouse IgG mAbs (1:20,000, Cat. No.: SC-2354; Santa Cruz Biotechnology).

### 2.7 Nano-flow cytometry for particle size measurements

A silica nanosphere cocktail (SiNP) (Cat. No.: S16M-Exo; NanoFCM Inc., Xiamen, China) containing a mixture of 68, 91, 113, and 155 nm silica beads was used for instrumental calibration and standard curve fitting. EP isolates and SiNP were analyzed with a nano-flow cytometer (nFCM) (NanoFCM Inc.) with the following parameters: sample volume, 100 μL; boosting time, 1 min; sampling, small signal; and sampling pressure, 1.0 kPa. Standard curve fitting and EP particle size were determined with the NF Profession software Version 1.0 (NanoFCM Inc.).

### 2.8 PacBio single molecule real-time (SMRT) sequencing

The PRTI region was amplified from HIV-1 DNA, and to distinguish sequences from different donors, the second-round PCR primers were barcoded. Purified DNA samples were subjected to library construction and SMRT analysis performed by Wuhan Frasergen Bioinformatics Co., Ltd. (Wuhan, China) with the PacBio Sequel platform (PacBio, Menlo Park, CA, USA). High-fidelity (HiFi) reads were generated in the PacBio circular consensus sequencing (CCS) mode with a minimum threshold on their quality (Q30).

The HiFi reads were analyzed with Lima software version 2.7.1 (https://lima.how/) to demultiplex and remove barcodes and primers. Sequences were aligned to the HIV-1 HXB2 strain reference sequence by using the Minimap2 software version 2.24 [25], and the alignment results were stored in .bam files. The Samtools software version 1.10 was used to quantify sequences and to transform .bam files into .fastq files [26]. These .fastq files were uploaded to the Stanford University HIV Drug Resistance Database Program: sequence reads analysis (https://hivdb.stanford.edu/hivdb/by-reads/) for DRM identifications.

### 2.9 Statistical analysis

To analyze the enrichment of HIV-1 RNA before and after EP-HIV-1 co-isolation, a non-parametric Wilcoxon signed-rank test was applied per D'Agostino and Pearson's omnibus normality test. Receiver operating characteristic (ROC) curve analysis was used to determine optimal cut-off values for dominant sequences identified through SMRT sequencing using ‘pROC’ package (version 1.19.0.1) in R. All statistical analyses were performed using R (Version 4.4.1), with statistical significance defined at *P* < 0.05 and Bonferroni correction for multiple comparisons was applied.

## 3 Results

### 3.1 EP-HIV-1 co-isolation enhances DRT at HIV-1 RNA level

**Fig. 2:**
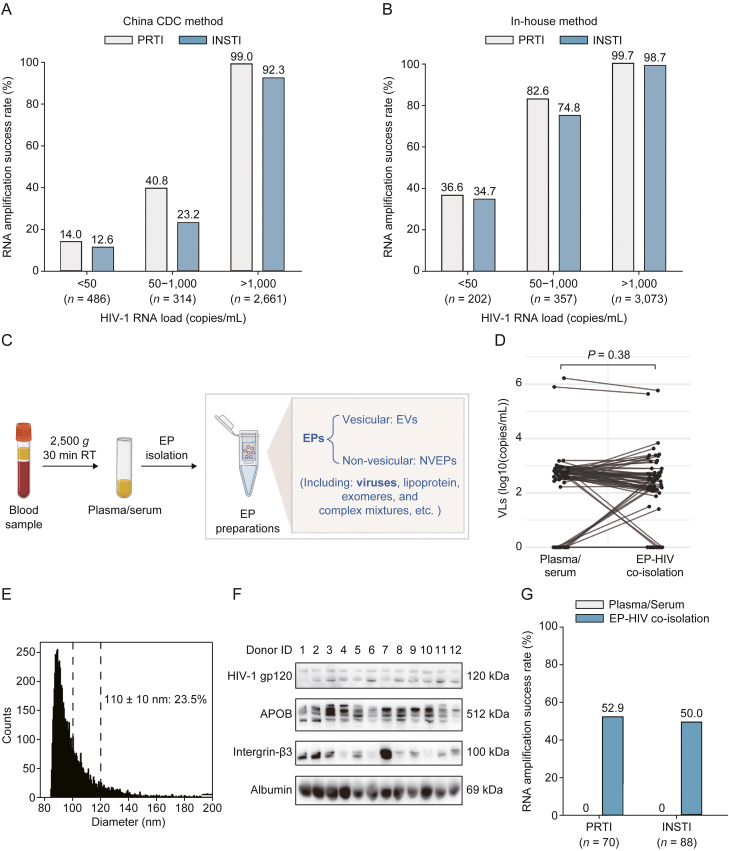
Enhancing human immunodeficiency virus type 1 (HIV-1) RNA drug resistance testing (DRT) through extracellular particle (EP)-HIV-1 co-isolation strategy. (A,B) Comparative analysis of amplification success rates: China CDC method (A) and in-house method (B). (C) Illustration of EP-HIV-1 co-isolation process. (D) Paired comparison of HIV-1 RNA viral loads (VLs) before and after EP co-isolation. VLs of each group obey normal distribution, while the mean differences of the two groups were non-normally distributed. Non-parameter paired *t*-test was employed (*n* = 75). (E) Size distribution of EPs determined by NanoFCM Inc. (F) Immunoblotting (IB) analysis. (G) Success rate of EP-HIV-1 co-isolation-based DRTs with samples that failed to be amplified from serum/plasma. China CDC: Chinese Center for Disease Control and Prevention; PRTI: protease and reverse transcriptase inhibitor; INSTI: integrase strand transfer inhibitor; RT: room temperature; EVs: extracellular vesicles; NEVPs: non-EV particles; APOB: apoprotein B.

To overcome the challenge of inefficient HIV-1 RNA amplification in LLV samples or those with residual viremia, we implemented a sequential two-stage strategy: initial primer optimization to improve amplification efficiency (in-house method), followed by EP co-isolation to enrich HIV-1 viral particles in samples that remained refractory to amplification. Similar to previously reported results, the China CDC method demonstrated a high success rate (99.0%) for the PRTI region in plasma/serum samples with a VL exceeding 1,000 copies/mL (Fig. 2A). However, the amplification success rates were remarkably lower (12.6%–40.8%) for samples with LLV (VL between 50 and 1,000 copies/mL) or residual viremia (VL < 50 copies/mL) (Fig. 2A). In comparison, with the in-house method the success rate for LLV samples increased to >74.8%, and for samples with VL < 50 copies/mL, the success rate rose to >34.7% (Fig. 2B). Furthermore, for samples with VL > 1,000 copies/mL, the amplification success rate for the INSTI region improved from 92.3% to 98.7% (Figs. 2A and B).

To further improve the success rate of RNA DRT, we established an ultrafiltration-based enrichment approach to co-isolate HIV-1 viral particles with EP (Fig. 2C). Firstly, we performed EP isolation on 75 plasma/serum samples. No significant differences in HIV-1 RNA VL were observed between the plasma/serum samples and their paired EP isolates (*P* = 0.38) (Fig. 2D). This observation suggested that the majority of HIV-1 RNA in plasma/serum could be effectively recovered through the EP isolation process. Using nFCM analysis, we observed that approximately 23.5% of EPs were within the diameter range (110 ± 10 nm) of HIV-1 viral particles (Fig. 2E). IB analysis revealed that the plasma/serum EP isolates exhibited the all-EV marker integrin β3, as well as the co-isolation contaminants such as albumin and APOB (Fig. 2F). Moreover, all EP isolates were tested positive for HIV-1 gp120 (Fig. 2F). Collectively, these findings strongly support the notion that HIV-1 viral particles can be co-isolated with EPs from plasma/serum samples.

Among the samples that failed to amplify using either the China CDC method or our in-house method, 52.9% (37/70) and 50.0% (44/88) achieved successful amplification through the EP-HIV-1 co-isolation process for the PRTI and INSTI regions, respectively (Fig. 2G).

### 3.2 High reproducibility of HIV-1 RNA and DNA DRTs

To further systematically evaluate the reproducibility between RNA and DNA DRTs, we analyzed DRM patterns across different VL strata, assessing DNA DRT amplification efficiency and determining the concordance between paired RNA and DNA DRT results. We observed that DRMs were prevalent across a broad VL range, spanning from VL < 50 to VL > 1,000 copies/mL (Fig. S1). In general, DRM distributions were similar among participants with VL > 1,000 copies/mL and LLV, namely the proportions of those with DRMs were ∼3.0% for PIs, ∼18.0% for NRTIs, ∼20.0% for NNRTIs, and ∼1.0% for INSTIs (Fig. S1). Notably, in participants with VL < 50 copies/mL, DRMs were detected in 0.7% for PIs, 14.1% for NRTIs, 17.6% for NNRTIs, and 0.8% for INSTIs (Fig. S1).

**Fig. 3:**
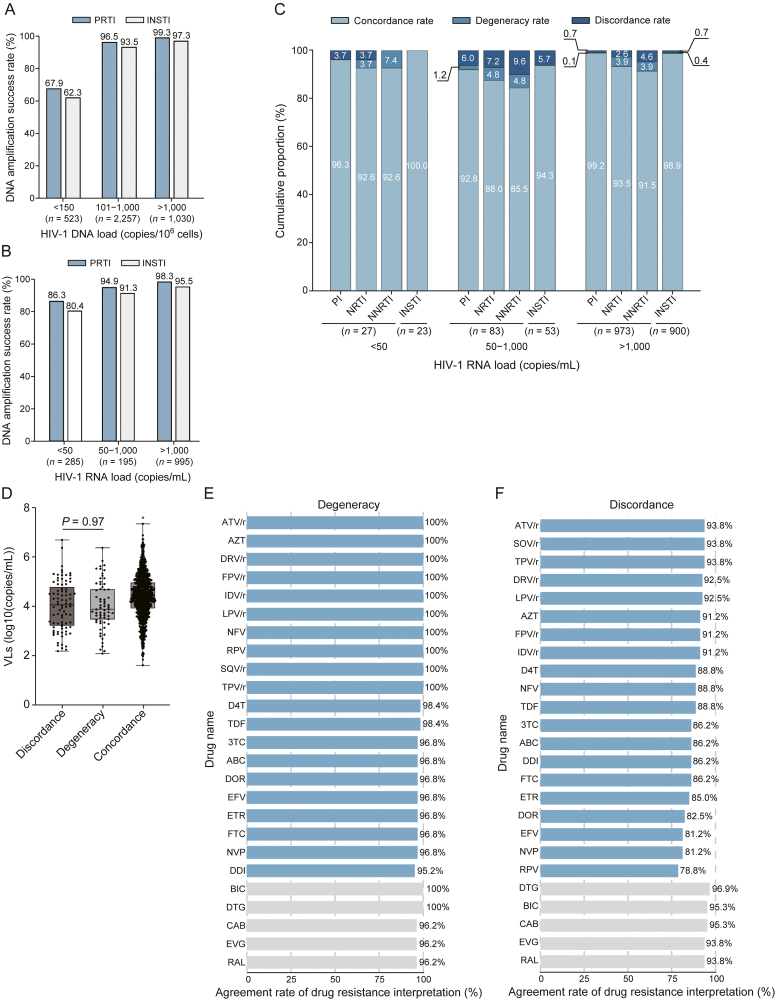
High reproducibility of human immunodeficiency virus type 1 (HIV-1) RNA and DNA drug resistance testing (DRT). (A, B) Amplification success rates at the HIV-1 DNA level stratified by HIV-1 DNA loads (A) and plasma HIV-1 RNA viral loads (VLs) (B). (C) Cumulative proportion of concordant, degenerate and discordant results comparing DRTs at HIV-1 DNA and RNA levels. Concordance and discordance refer to identical and completely different results that are obtained from the two DRTs, respectively. Degeneracy refers to that multiple drug resistance mutations (DRMs) can be detected at HIV-1 DNA level, but they include all DRMs detected from HIV-1 RNA. (D) VL distribution among discordance, degeneracy, and concordance samples. (E, F) Agreement rate of DNA and RNA DRT-derived drug resistance interpretation: degeneracy (E) and discordance (F). Here, the agreement rate was calculated based on the Stanford penalty scores for sequences against 25 antiviral therapy (ART) drugs. Sequences with DRMs were interpreted to be susceptible (<15) or resistant (≥15) based on their penalty scores against certain drugs, while the agreement rate is defined as the percentage of matching resistance interpretations between HIV DNA and RNA DRTs. ^∗^ Represents significant difference from others, Student's *t*-test. PRTI: protease and reverse transcriptase inhibitor; INSTI: integrase strand transfer inhibitor; PI: protease inhibitor; NRTI: nucleoside reverse transcriptase inhibitor; NNRTI: non-NRTI; ATV/r: atazanavir/ritonavir; AZT: zidovudine; DRV/r: darunavir/ritonavir; FPV/r: fosamprenavir/ritonavir; IDV/r: indinavir/ritonavir; LPV/r: lopinavir/ritonavir; NFV: nelfinavir; RPV: rilpivirine; SQV/r: saquinavir/ritonavir; TPV/r: tipranavir/ritonavir; D4T: stavudine; TDF: tenofovir disoproxil fumarate; 3TC: lamivudine; ABC: abacavir; DOR: doravirine; EFV: efavirenz; ETR: etravirine; FTC: emtricitabine; NVP: nevirapine; DDI: didanosine; BIC: bictegravir; DTG: dolutegravir; CAB: cabotegravir; EVG: elvitegravir; RAL: raltegravir.

We further evaluated the performance of DNA DRTs with 3,810 whole blood samples. For samples exceeding the LOQ of HIV-1 DNA, over 93.5% achieved successful amplification for both PRTI and INSTI regions, with respective success rates from 93.5% to 99.3% (Fig. 3A). In contrast, samples with the HIV-1 DNA load below the LOQ, lower success rates at less than 70.0% were observed (Fig. 3A). When stratifying samples based on VL, 94.9% and 91.3% of LLV samples (*n* = 195) were successfully amplified for the PRTI and INSTI regions, respectively (Fig. 3B). Notably, even among samples with VL < 50 copies/mL (*n* = 285), over 80.4% achieved successful amplification with HIV-1 DNA (Fig. 3B).

Among all tested samples, 1,475 had paired RNA and DNA DRT results, in which the median HIV-1 DNA loads were 279, 402, and 722 copies/10^6^ cells in the three strata of VLs, respectively (Fig. S2). These RNA/DNA DRT results could be categorized into three scenarios: concordance (identical DRMs detected from both DRTs), discordance (completely different DRMs detected), and degeneracy (multiple DRMs detected in DNA but inclusive of all RNA-derived DRMs). Examples of degeneracy and discordance DRT results can be found in Table 1, while all such DRT results are provided in Tables S2 and S3. Overall, we observed that RNA DRT-derived DRMs were predominantly reproducible by DNA DRTs, with combined concordance and degeneracy rates (C/D rates) ranged from 90.4% to 100% in different gene regions (Fig. 3C). Specifically, C/D rates were 98.8% for the PI region (*n* = 1,083) and 99.1% for the INSTI region (*n* = 976), with discordance rates of 1.2% and 0.9%, respectively (Fig. 3C). In contrast, higher discordance rates were observed for the NRTI (2.8%) and NNRTI (4.9%) regions (Fig. 3C). Detailed discordance data are available in Table S2.

**Table 1:** Examples of drug resistance testing (DRT) result classifications regarding degeneracy and discordance.

Sample ID	NRTIs		Category
	RNA	DNA	
45	M184V	M184MV	Degeneracy
316	M41L and K219E	M41ML, K70KT, and K219KE	Degeneracy
274	K70KE and M184V	M184MV	Discordance
811	M184V	–	Discordance
1467	–	M184I	Discordance

−: wild type. NRTIs: nucleoside reverse transcriptase inhibitors.

To elucidate the factors underlying the inconsistency between RNA and DNA-based DRT results, we assessed their association with VL and impact on clinical interpretations. Patients in the discordance and degeneracy groups exhibited significantly lower plasma VLs than those in the concordance group (*P* < 0.01; Fig. 3D), suggesting that stochastic sampling effects during SS contribute to sequence-level discrepancies at low VLs. We further evaluated whether these sequence differences affected clinical resistance interpretations. Based on Stanford penalty scores across 25 ART drugs, the agreement rate of drug resistance interpretations between DNA and RNA DRTs within the degeneracy group was 98.3% ± 1.7% (range: 95.2%–100%) (Fig. 3E). Even within the discordance group, the mean agreement rate remained high at 89.5% ± 5.0% (Fig. 3F), with only four NNRTIs, showing agreement below 85% (DOR, 82.5%; EFV, 81.2%; NVP, 81.2%; and RPV, 78.8%). These findings indicate that although sequence-level discordance increases at lower VLs, the core clinical resistance interpretation remains predominantly consistent, underscoring the reliability of DNA DRT for guiding therapeutic decisions.

### 3.3 Experimental modeling of dominant sequences in HIV-1 DNA DRTs

To determine the cut-off value for dominant sequences that were preferentially sequenced by SS in DNA DRT, we analyzed the PRTI region amplicons through SMRT sequencing using 12 LLV whole blood samples. A total of 505,471 HiFi reads were obtained and demultiplexed to individual participants. Based on the Stanford University HIV Drug Resistance Database, 72 amino acid (AA) positions were included in this analysis, and HiFi reads with 2407 positioned AAs were analyzed.

**Fig. 4:**
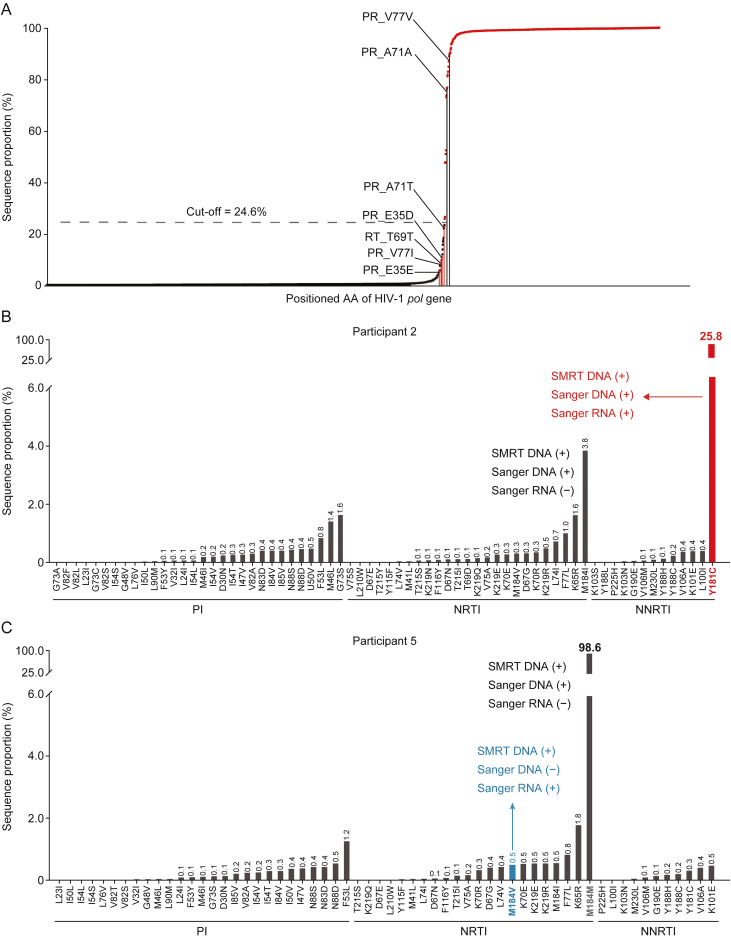
Experimental modeling of dominant sequences in human immunodeficiency virus type 1 (HIV-1) DNA. (A) Sequence proportion distribution based on positioned amino acid (AA). For a certain sequence containing a particular positioned AA, sequence proportion refers to the ratio of the copy number of sequences containing this AA to the copy number of all sequences that cover this AA position in a certain participant. The 2,407 positioned AAs are located in the *x*-axis based on their sequence proportions from low to high. The red dots indicate that a positioned AA can be detected in either single molecule real-time (SMRT) or Sanger sequencing (SS), and the black dots indicate those positioned AAs that can only be detected by SMRT sequencing but not by SS. Mutations are denoted by the wild-type AA, the position in the protein or region sequence, and the mutant AA (e.g. PR_V77I). (B) Example of dominant sequences in HIV-1 DNA that can be detected by SMRT sequencing and SS drug resistance testings (DRTs) with either HIV-1 DNA or RNA. (C) Example of the HIV-1 RNA DRT detected drug resistance mutation (DRM) that corresponds to the minor SMRT sequence. PI: protease inhibitor; NRTI: nucleoside reverse transcriptase inhibitor; NNRTI: non-NRTI.

For a given sequence containing a specific positioned AA, the sequence proportion was defined as the ratio of the copy number of sequences containing this AA to the total copy number of sequences covering this AA position within a certain sample (Fig. 4A). ROC curve analysis was then performed to evaluate the relationship between sequence proportion and detectability in SS. The sequence proportion corresponding to a 24.6% threshold yielded the maximum Youden index and was determined as the optimal cut-off for defining dominant sequences. With the sample size of 2,407 positioned AAs, the statistical power of the obtained threshold of 24.6% was >99.9% at a significance level of alpha = 0.05 (Table S4).

We next conducted a comparative analysis at the individual participant level, examining SMRT sequencing results alongside paired RNA and DNA DRT outcomes. Detailed sequence proportions and corresponding positioned AA information are presented in Table S5. Among 12 participants, 10 showed complete concordance between the dominant HIV-1 DNA sequences identified via SMRT and the DRM results from both RNA and DNA DRTs. For instance, Participant 2 exhibited a dominance sequence carrying the DRM Y181C with a sequence proportion of 25.8% per SMRT sequencing, which was consistently detected in both RNA and DNA DRTs (Fig. 4B). In contrast, two participants demonstrated discordant results. Participant 5, for example, displayed a dominant wild-type sequence at the M184 position, accounting for 98.6% of SMRT sequences, aligning with DNA DRT findings. However, the minor SMRT sequence (0.5%) corresponding to M184V was only detected in RNA DRT (Fig. 4C). A similar case was observed for Participant 3, as detailed in Fig. S3.

## 4 Discussion

In this study, we present strong evidence supporting the application of HIV-1 DNA DRT in clinical practice. Our findings specifically addressed concerns regarding the interpretation of DRMs detected in HIV-1 DNA through SS. These findings have substantial implications for optimizing HIVDR surveillance strategies and clinical decision-making processes, thereby potentially enhancing the monitoring and/or management of PLWHs.

In this study, we tried to increase the RNA amplification rates in diverse VL states, which served as an important basis for the reproducibility analysis on RNA and DNA DRTs. Especially, we introduced an EP-HIV co-isolation method that could sufficiently recover serum/plasma HIV-1 RNA. In the field of extracellular vesicles (EVs), the term “EPs” serves as an umbrella category encompassing all particles located outside the cell, including EVs and non-vesicular EPs (NVEPs) [27]. This terminology is adopted due to the current absence of universally accepted markers for establishing EV purity [27,28]. EPs can be stratified based on physical characteristics such as size; for instance, small EVs (sEVs) are defined as an EP subset with diameters less than 200 nm [27]. Notably, the mean diameter of HIV-1 viral particles is well-established to be approximately 110 ± 8 nm [29], which is comparable to the size range of sEVs and specific NVEPs like APOB-lipoproteins, albumin aggregates, and exomeres [27]. This size similarity led us to hypothesize that HIV-1 viral particles could be co-isolated alongside EPs from plasma samples. We further showed that this approach was of remarkable capacity to improve the success rates of RNA amplifications. To understand such improvement, we have previously shown that this approach detects residual HIV-1 virus in approximately 50.0% of plasma samples with undetectable VLs [23]. Comparably, studies from other groups have also shown that viral enrichment with ultracentrifugation can increase the HIV-1 RNA amplification success rates [30,31]. Taking these together, EP-HIV co-isolation tends to decomplex sample composition that leads to improved PCR performance.

Mixed codons have been frequently observed in HIV-1 genotyping through SS, which are proven to be indicative of the proportional composition of viral quasispecies within an individual [32,33]. These mixed codons can be clinically interpreted in conjunction with a patient's medication history and adherence data [1]. The DRMs identified in DNA DRTs may originate from one of three proposed pathways: ART selection pressure [1], archived quasispecies from repeated exposures [14], or apolipoprotein B messenger RNA (mRNA) editing enzyme, catalytic polypeptide-like (APOBEC)-mediated G-to-A hypermutation [34]. Notably, baseline minority variants in plasma HIV-1 RNA, particularly those conferring NNRTIs resistance, have been significantly associated with an elevated risk of virologic failure [35]. Furthermore, meta-analytic data indicate that resistance trends observed in HIV-1 proviral DNA generally correlate with those derived from HIV-1 RNA [36]. Thus, minority but replication-competent HIV-1 DNA variants can be interpreted as a transitional state between dominant DNA DRMs and their RNA counterparts (see Fig. 4B for an example), aiding in the understanding of viral evolutionary dynamics under selective pressure. This conceptual framework supports the clinical interpretation of degeneracy results, in which multiple DNA variants may be detected either by exceeding the detection threshold of SS or through stochastic sampling effects. Consequently, monitoring temporal changes in minor DNA variants may hold predictive value for assessing the risk of future drug resistance and/or treatment failure. These considerations reinforce our argument in this study to use C/D rates to reflect the reproducibility of DRM detections in RNA and DNA DRTs. We showed that C/D rates were >95.4% for samples with VL > 1,000 copies/mL and VL < 50 copies/mL, higher than those in LLV samples (90.4%–94.3%). In addition, our findings align with the well-documented higher resistance barriers for PIs and INSTIs, compared to NRTIs and NNRTIs, which have a longer history of clinical use [37]. Furthermore, based on Stanford penalty score analysis, this study demonstrated high agreement in drug resistance interpretations between RNA and DNA DRT results within both the degeneracy and discordance groups. It should be noted, however, that for patients receiving NNRTIs of DOR, EFV, NVP, or RPV, which exhibited relatively lower drug resistance agreement rates, the use of highly sensitive RNA DRT (e.g., employing an EP-HIV co-isolation method) in parallel with DNA DRT is recommended.

In SS, sampling of HIV-1 sequences is a probabilistic process influenced by the proportional representation of viral quasispecies. The mathematical modeling of dominant sequence proportions provides a theoretical foundation for interpreting SS-based DRT results. Previous studies have characterized this phenomenon. Larder et al. [38] have employed HIV DNA standards to simulate mixed viral populations, demonstrating that SS preferentially detects mutant sequences that constitute ≥25.0% of the total population. Similarly, Hebert et al. [39] have estimated the proportion of DRMs using PacBio SMRT sequencing, incorporating known PCR errors of the Platinum Taq enzyme, showing that dominant sequences typically represent approximately 40.0% of the viral quasispecies. In our current study, we established a cut-off proportion for dominant sequences in HIV-1 DNA samples at 24.6%, a value comparable to prior estimates obtained using SS-based RNA sequencing. Furthermore, our findings demonstrated that in 10 out of 12 (83.3%) LLV samples, the dominant HIV-1 DNA sequence matched the SS results from either HIV-1 RNA or DNA, which is helpful to understand the high reproducibility of RNA and DNA DRTs. We also noted that in two cases, DRMs detected from RNA DRTs were corresponding to minor sequences of DNA. Such an observation suggests that one of the possible explanations of discordant RNA and DNA DRT results is the stochastic sampling per SS.

We and others have demonstrated that DNA DRT plays a significant role in informing treatment decisions for PLWHs. For instance, Alagaratnam et al. [40] have highlighted the utility of HIV-1 DNA DRT in determining the suitability of long-acting injectable antiretroviral therapies, such as CAB/RPV. Our previous studies have shown that in cases where HIV-1 RNA DRTs fail, adjusting ART regimens based on DNA DRTs can lead to successful virologic suppression in LLV patients [22,41]. In addition, Armenia et al. [42] found that in virologically suppressed patients who experienced treatment switching, baseline DRMs detected HIV-1 DNA was an important predictor for virologic rebound.

Although a comprehensive cost-effectiveness analysis comparing RNA and DNA DRTs was not conducted in this study, several inherent advantages of DNA DRTs can be highlighted to support their adoption in resource-limited settings. First, the DNA DRT protocol omits the reverse transcription step required for RNA DRTs, which correspondingly reduces reagent costs by 47% and shortens hands-on labor time during PCR amplification by 14.3%. Second, DNA DRTs can be performed on more accessible sample types, such as whole blood and dry blood spots, thereby eliminating the need for plasma separation, a process that necessitates centrifugation and cold chain logistics for RNA DRTs. Furthermore, the superior chemical stability of HIV-1 DNA compared to RNA remarkably relaxes the stringent conditions required for sample transportation and storage. These operational and practical benefits favor DNA DRT as a viable and efficient alternative for drug resistance monitoring in settings where cost, sample stability, and procedural simplicity are critical constraints.

This study has several limitations that should be considered when interpreting the results. First, the viral subtypes analyzed were predominantly CRF01_AE and CRF07_BC, which are the dominant subtypes in China. Consequently, the dataset contains limited information on other subtypes that are prevalent in different geographical regions, such as Subtype B in America and Europe, and Subtype C in Africa, which warrants further multicenter validation to generalize our findings to those populations. Second, the cross-sectional design of this study does not include follow-up data, which precludes the assessment of long-term clinical outcomes following therapeutic decisions based on the DNA DRT results. Third, although power calculation justified the confidence in the identified dominant sequence threshold of 24.6%, the donor sample size (*n* = 12) used for this specific analysis is relatively small. This may limit the robustness of the threshold when applied to populations with more complex subtype compositions.

**Fig. 5:**
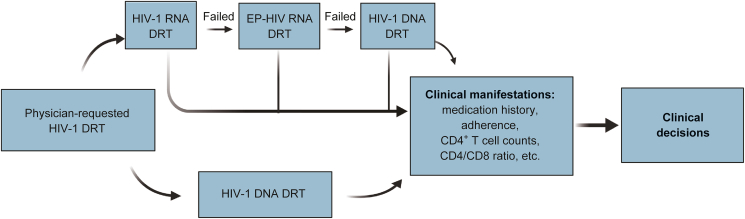
Proposed strategy to integrate human immunodeficiency virus type 1 (HIV-1) DNA drug resistance testing (DRT) in clinical decision-making process. EP: extracellular particle.

In summary, this study provides robust evidence supporting the clinical utility of DNA DRT as a reliable supplement or alternative to RNA-based genotyping, particularly for patients with low-level or suppressed viremia where conventional RNA testing often fails. As such, an organized strategy to integrate DNA DRT into clinical decision-making processes to enhance the precision and reliability of treatment recommendations can be proposed (Fig. 5). First, physician-requested HIV-1 DRTs can be sequentially performed at HIV-1 RNA and DNA levels, which the EP-HIV-1 co-isolation can be performed to increase the success amplification rate of HIV-1 RNA. Second, based on the high C/D rates demonstrated in this study, DNA DRT can be employed as a cost-effective alternative to RNA DRT. Both workflows will render useful information for clinical decisions, especially when considering medication history and adherence, as well as other manifestations such as CD4^+^ T cell counts and CD4/CD8 ratio (Fig. 5).

## 5 Conclusions

This nationwide study analyzed a large-scale cohort of patients across diverse VL strata, and we demonstrated that DNA DRT maintains high reproducibility with RNA DRT in detecting DRMs, with combined C/D rates exceeding 90% across major antiretroviral drug classes. Critically, the clinical interpretation of DNA-based results showed strong agreement with RNA-derived profiles, reinforcing the utility of proviral DNA genotyping for informing treatment decisions. We experimentally determined the 24.6% dominant sequence threshold using single-molecule sequencing, which quantified the detectability limit of SS and provided a mechanistic basis for interpreting reproducibility between RNA and DNA DRT results. Moreover, the successful application of EP-HIV co-isolation and optimized primer designs significantly enhanced RNA detection sensitivity, providing complementary strategies for difficult-to-amplify samples. These findings support the integration of DNA DRT into standard HIV management protocols, especially in resource-limited settings or for patients with LLV. Future studies should focus on validating these approaches across diverse HIV-1 subtypes and establishing the long-term clinical impact of DNA-guided treatment strategies.

## CRediT authorship contribution statement

**Caihong Wu:** Writing – review & editing, Writing – original draft, Validation, Methodology, Investigation, Formal analysis, Data curation. **Limin Zhang:** Writing – review & editing, Writing – original draft, Visualization, Methodology, Investigation, Formal analysis, Data curation. **Zhong Chen:** Writing – original draft, Resources, Project administration, Investigation, Funding acquisition, Data curation, Conceptualization. **Wencui Ma:** Methodology, Investigation, Formal analysis. **Yanhua Fu:** Validation, Resources, Investigation. **Ke Yang:** Validation, Resources, Investigation. **Mei Liu:** Validation, Resources, Investigation. **Yanjun Li:** Validation, Resources, Investigation. **Xiaohong Chen:** Validation, Resources, Investigation. **Mingjie Hou:** Validation, Resources, Investigation. **Min Liu:** Validation, Resources, Investigation. **Aihua Deng:** Validation, Resources, Investigation. **Qingxia Zhao:** Validation, Resources, Investigation. **Lukun Zhang:** Validation, Resources, Investigation. **Quan Wang:** Validation, Resources, Investigation. **Jun Peng:** Validation, Project administration, Investigation. **Yongli Li:** Software, Formal analysis, Data curation. **Keji Deng:** Validation, Project administration, Investigation. **Jingsong Bai:** Validation, Resources, Investigation. **Hai Long:** Validation, Resources, Investigation. **Yaokai Chen:** Validation, Resources, Investigation. **Hui Wang:** Validation, Resources, Methodology. **Yun He:** Validation, Resources, Investigation. **Jin Li:** Validation, Resources, Investigation. **Jiahui Guo:** Writing – review & editing, Formal analysis. **Bianchuan Cao:** Validation, Resources, Investigation. **Yizhi Cui:** Writing – review & editing, Visualization, Formal analysis. **Min Wang:** Validation, Resources, Project administration, Investigation. **Tuofu Zhu:** Writing – review & editing, Writing – original draft, Project administration, Methodology. **Jun Yao:** Writing – review & editing, Validation, Methodology, Investigation. **Tong Wang:** Writing – review & editing, Writing – original draft, Supervision, Project administration, Funding acquisition, Conceptualization.

## Data availability

All sequences data in this study were submitted to HIV Gene Sequences Database, China (https://nmdc.cn/hiv/) (Submission No.: SUB1720661380069). These data will be shared with researchers who provide a methodologically sound application proposal. Application proposals should be sent to tongwang@email.edu.cn.

## Declaration of competing interest

The authors declare that they have no known competing financial interests or personal relationships that could have appeared to influence the work reported in this paper. Authors Jun Peng, Keji Deng, and Tuofu Zhu served as Assistant President, Vice President, and Chief Scientist of Guangzhou SupBio Bio-Technologies and Science Co., Ltd., respectively; author Yongli Li served as Engineer of Dongguan Institute of Microscale and Precision Medical Measurement Co., Ltd. during the research. Moreover, Guangzhou SupBio Bio-Technologies and Science Co., Ltd. and Dongguan Institute of Microscale and Precision Medical Measurement Co., Ltd. have no relevant relationships or competing interests related to this research.
