# Supplementary material for: Large-scale evaluation of HIV-1 DNA drug resistance testing as a robust tool for clinical decision-making: A nationwide study in China
Source: J Pharm Anal. 2025 Dec 9;16(1):101513. doi: 10.1016/j.jpha.2025.101513 (PMC12924009; doi:10.1016/j.jpha.2025.101513)
Supplement: Multimedia component 1 [file mmc1.docx]

**Supplementary Materials**

**Large-scale Evaluation of HIV-1 DNA Drug Resistance Testing as** **a Robust Tool for Clinical Decision-Making: A Nationwide Study in China**

**
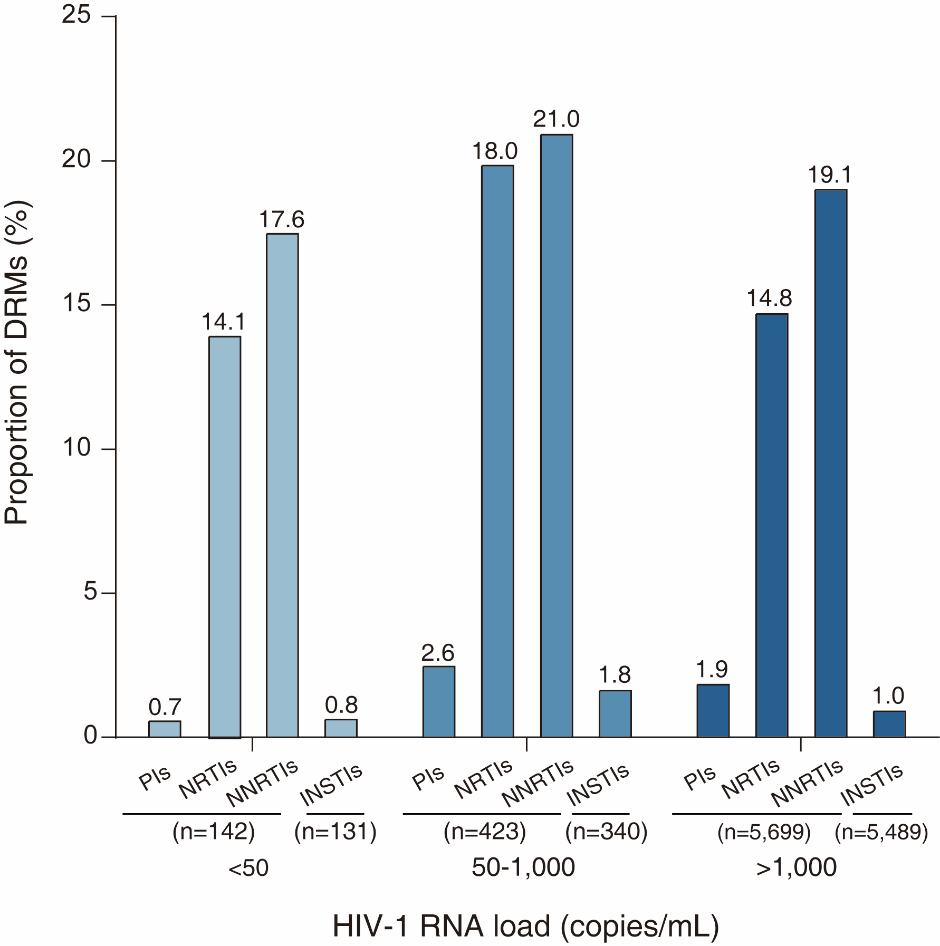
**

**Supplementary Figure S1.** HIVDR profiles stratified by plasma HIV-1 RNA viral loads.

**
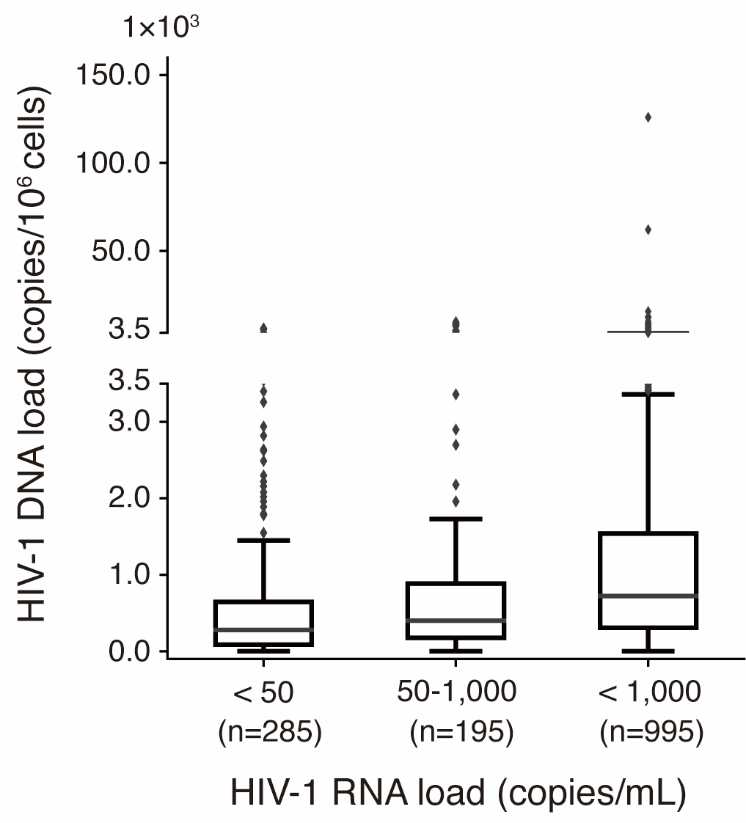
**

**Supplementary Figure S2.** HIV-1 DNA load distribution stratified by plasma HIV-1 RNA viral loads.

**
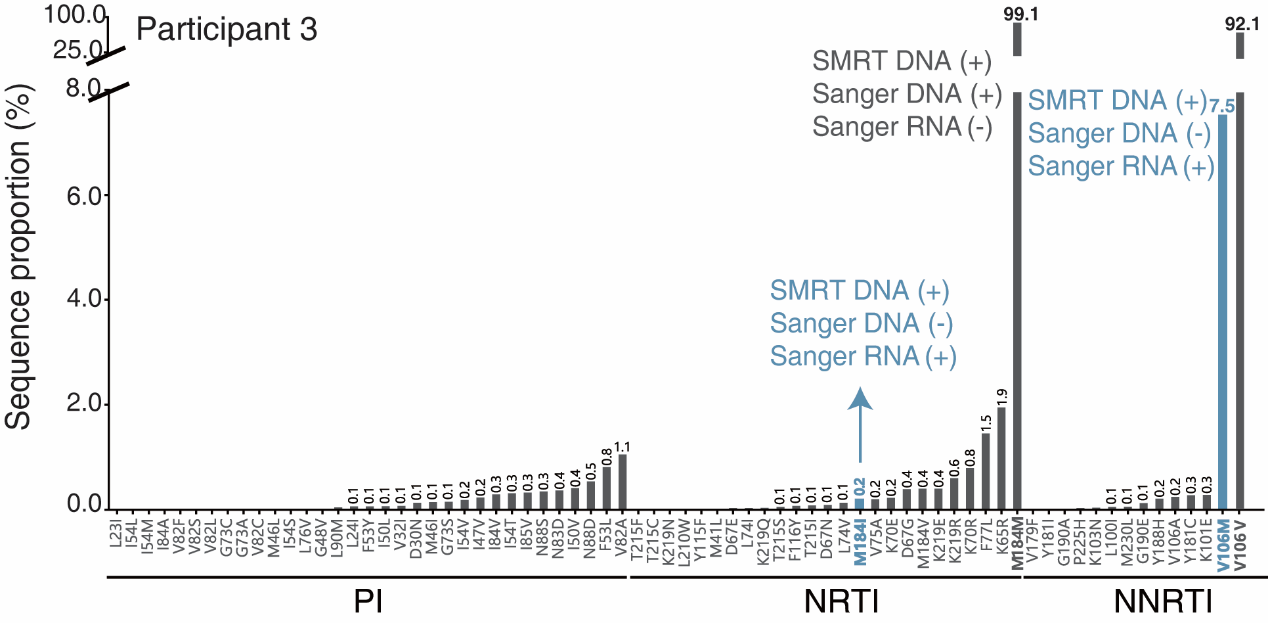
**

**Supplementary Figure S3.** The other example of the HIV-1 RNA DRT detected DRM that corresponds to the minor SMRT sequence.

**Supplementary Table S1.** Nucleotide sequences of optimized primers for in-house HIV-1 RNA/DNA DRT.

| Target Region | Primer Name | Sequence (5' → 3') |
| --- | --- | --- |
| PRTI | SEQ_1 | CAGTAGGAGAAATTTATAAAAGATGGAT |
| PRTI | SEQ_2 | TGTTCATTTCCTCCAATTCCTTT |
| PRTI | SEQ_3 | GGAAAGGAAGGACACCAAATGA |
| PRTI | SEQ_4 | TAAATTTAGGAGTCTTTCCCCATATTAC |
| INSTI | SEQ_5 | CAGAAAGCATAGTAATATVGGG |
| INSTI | SEQ_6 | GTCCTGCTTGATATTCACACC |
| INSTI | SEQ_7 | ATGGGTACCAGCACACAAAGGAAT |
| INSTI | SEQ_8 | AAATGCCAGTCTCTTTCTCCTG |

**Supplementary Table S4.** Power analysis table.

|  | Power^b^ | Test Assumptions | | | | | |
| --- | --- | --- | --- | --- | --- | --- | --- |
|  |  | N1 | Std. Dev1 | N2 | Std. Dev2 | Mean Difference | Sig. |
| Test for Mean Difference^a^ | 1.000 | 847 | 8.62388 | 1560 | 3.29248 | 97.240 | .05 |
| a. Two-sided test. | | | | | | | |
| b. Based on noncentral t-distribution. | | | | | | | |
